# Supplementary material for: Seizure forecasting by tracking cortical response to electrical stimulation
Source: Epilepsia. 2025 Jul 15;66(10):3937–47. doi: 10.1111/epi.18518 (PMC12611449; doi:10.1111/epi.18518)
Supplement: Supplementary file 1 — Appendix S1. [file EPI-66-3937-s001.docx]

**Supplementary material**

**Supplementary Methods**

**Feature computation**

Cortical response to electrical stimulation was quantified using the variance $\sigma^{2}= \frac{1}{N-1}\sum_{i=1}^{N} \left( x_{i}-\bar{x} \right)^{2}$, where $N$ is the number of samples of the signal $x$, and autocorrelation $\rho_{\lambda}=\frac{1}{N-1}\frac{\sum_{i=1}^{N-\lambda} {(x}_{i}-\bar{x}) (x_{i+\lambda}-\bar{x})}{\sigma^{2}}$ as a function of the lag $\lambda.$The autocorrelation metric was computed as the width at the half maximum of the autocorrelation function [Maturana_2020]. When we considered in the analysis more than one signal, we computed the average of variance and autocorrelation across the analysed signals. For each value of variance and autocorrelation, we also computed its cumulative average across its previous 12 values that correspond to a one-hour interval (i.e., $12\times5\min=1hour$). Hence, for a given number of analysed iEEG signals (one, five or all), a given analysis window (one pre-stimulation window, three windows post the first stimulation, and two windows post the second stimulation) we obtained a feature vector, i.e., variance, autocorrelation, cumulative average of variance and cumulative average of autocorrelation.

Features in the 10min time interval before and after the seizure timestamp were removed from the analysis. In addition, all features were smoothed using a backward moving average filter with a 30min window length (i.e., average between the current and previous five points) to eliminate spontaneous fluctuations. Specifically, features were split in two windows that were separated at the timestamp of the seizure. Smoothing was performed in each window separately and features that corresponded to the first 30min of each window were discarded from the analysis to eliminate edge effects.

**Seizure forecasting metrics**

To quantify the performance of the seizure forecasting algorithm, we used previously described forecasting metrics [Cook_2013, Karoly_2017]. Specifically, we used Sensitivity ($S = TP/(TP+FN)$, where $TP$: seizures that occur when the alarm is on, $FN$: seizures that occur outside alarms), time spent in warning ($tiw$: the proportion of time that was spent in warning computed as the total number of points for which the alarm was on over the total number of points), Improvement over chance ($IoC = Sensitivity - tiw$) and Brier Skill Score that quantifies the improvement of the Brier score relative to a random reference ($BSS= 1-\frac{BS}{BS_{ref}}$ where BS is the Brier Score $BS =\frac{1}{n}\sum_{i=1}^{n} \left( f_{i}-o_{i} \right)^{2}, n$ is the number of forecasted points, $f_{i}$ forecasted probability of the $i^{th}$ forecasted point, $o_{i}$ observed value of the $i^{th}$ point (0 when the point had “inter-ictal” label and 1 when it had the “pre-ictal” label). $BS_{ref}$ was computed by randomly shuffling the probability forecasts 100 times and afterwards taking the average BSS value). When there is not improvement over reference then BSS tends to 0, when it is worse than reference it tends to $- \infty$ and to 1 when it is perfect.

**Supplementary Figures**


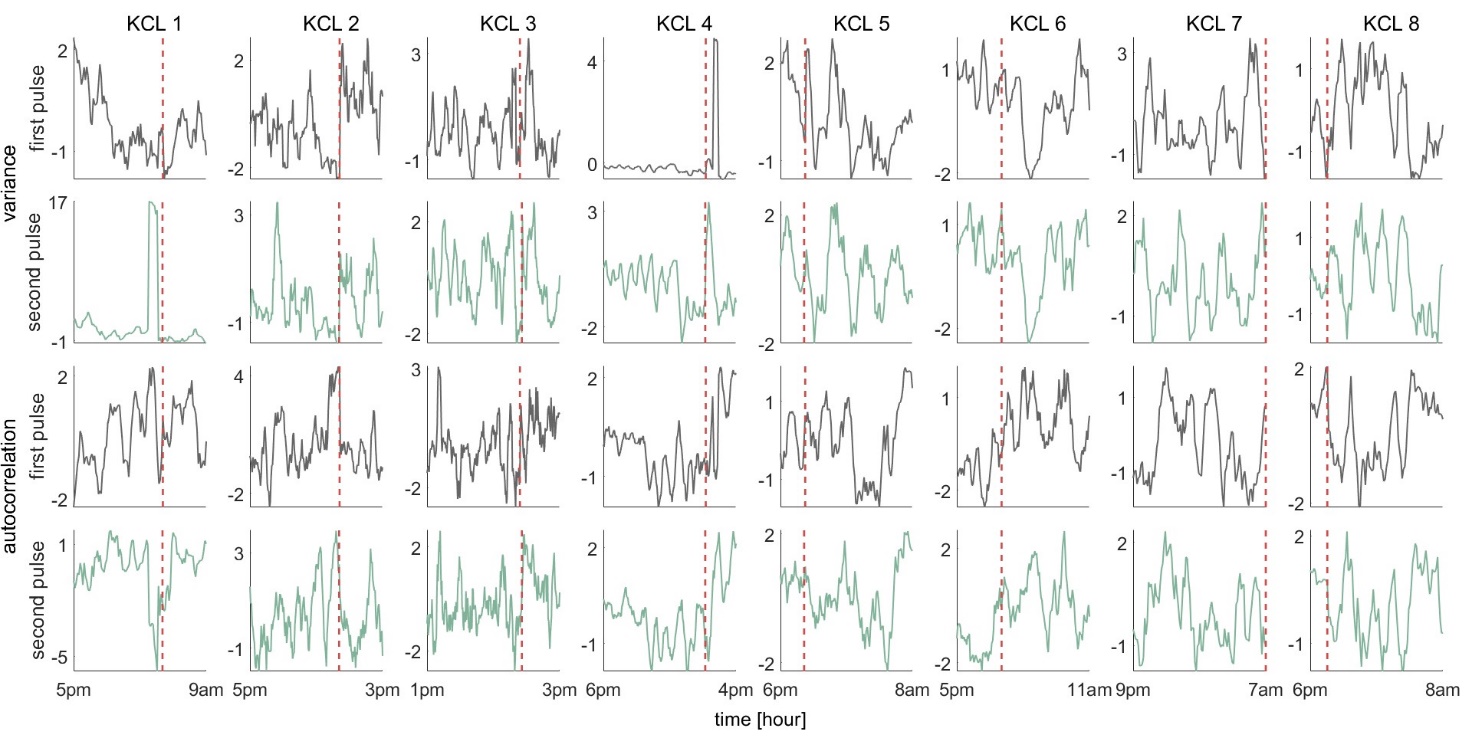


Figure S1: Temporal evolution of the features from the window 20ms to 100ms after the first stimulus (grey) and after the second stimulus (green). The vertical dashed line denotes the seizure onset.


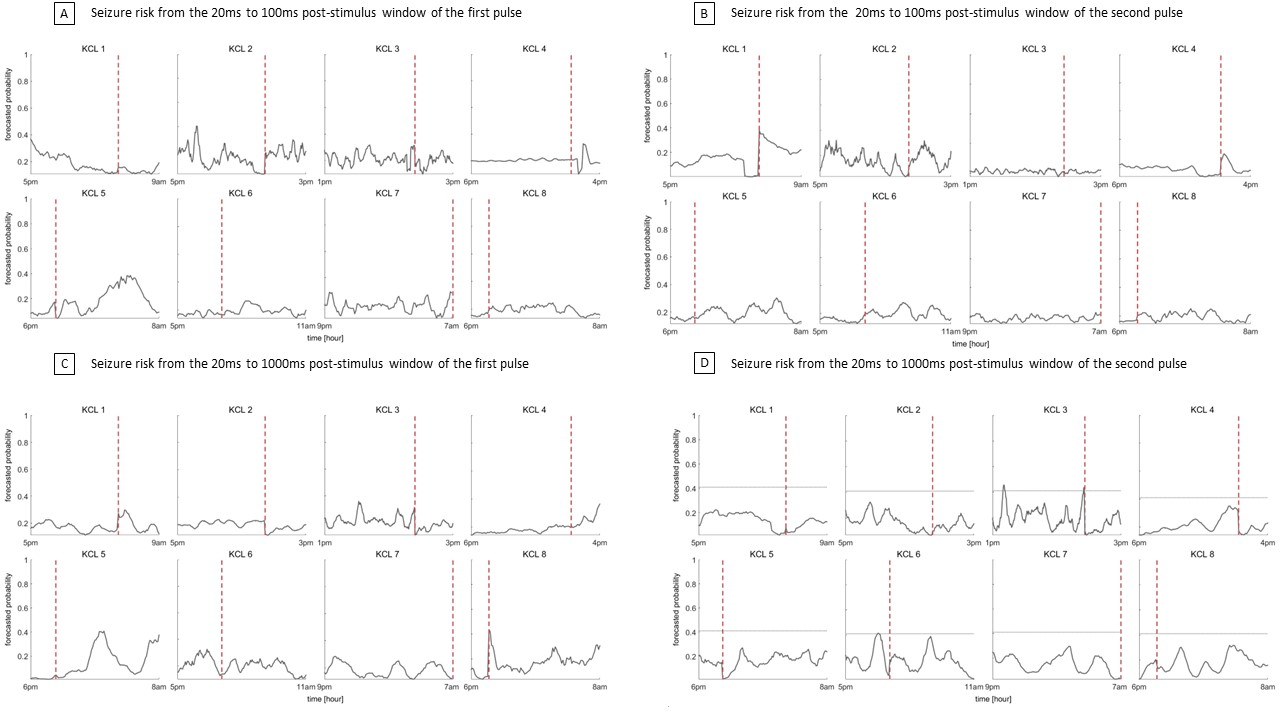


Figure S2: Seizure likelihood computed from the windows 20ms to 100ms and 20ms to 1000ms after the first (A, C) and second stimulus (B, D). The vertical dashed line denotes the seizure onset. All patients in panels A, B and C have zero Improvement over chance. The grid search (see Methods) in patients of panel D set the probability threshold (horizontal line) to 0.41 and the seizure occurrence period to 90min. Forecasting metrics in panel D: KCL3 (sensitivity:1, tiw: 0.08, IoC: 0.92, forecasting horizon: 17.7min, BSS: 0.03) and KCL 4 (sensitivity:1, tiw: 0.09, IoC: 0.9, forecasting horizon: 88.9 min, BSS: 0.07) respectively.


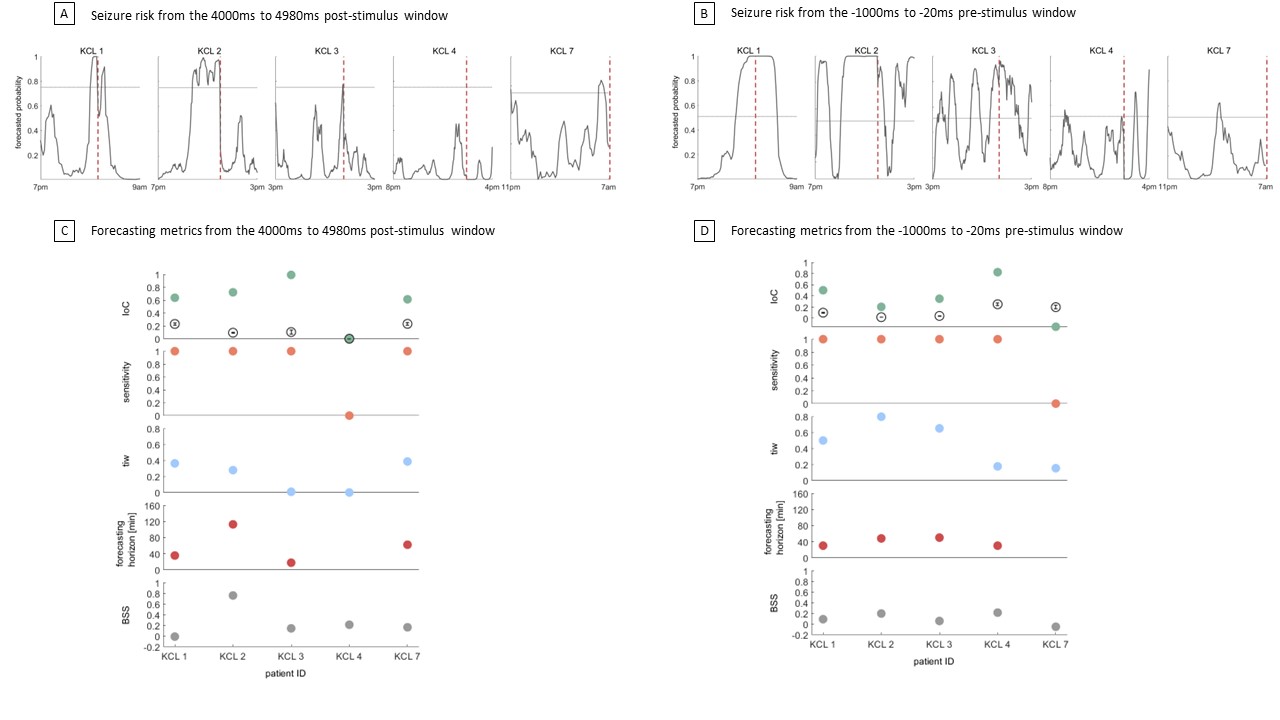


Figure S3: Seizure likelihood and forecasting metrics computed from the window 4000ms to 4980ms after the first stimulus (A, C) and the -1000ms to -20ms window prior to the first stimulus (B, D). Note that during the leave-one-subject-out approach no future information was used in the test patient. The vertical dashed line denotes the seizure onset. After grid search (see Methods) the probability threshold (horizontal line) and seizure occurrence period were set to 0.75; 120min (A) and 0.51; 60min(B) respectively. Gray unfilled circles in panels C and D denote the average IoC obtained from the shuffled forecasts across 100 runs, whilst error bars denote the standard error. Note that negative IoC values from the shuffled forecasts were set to zero prior to averaging.


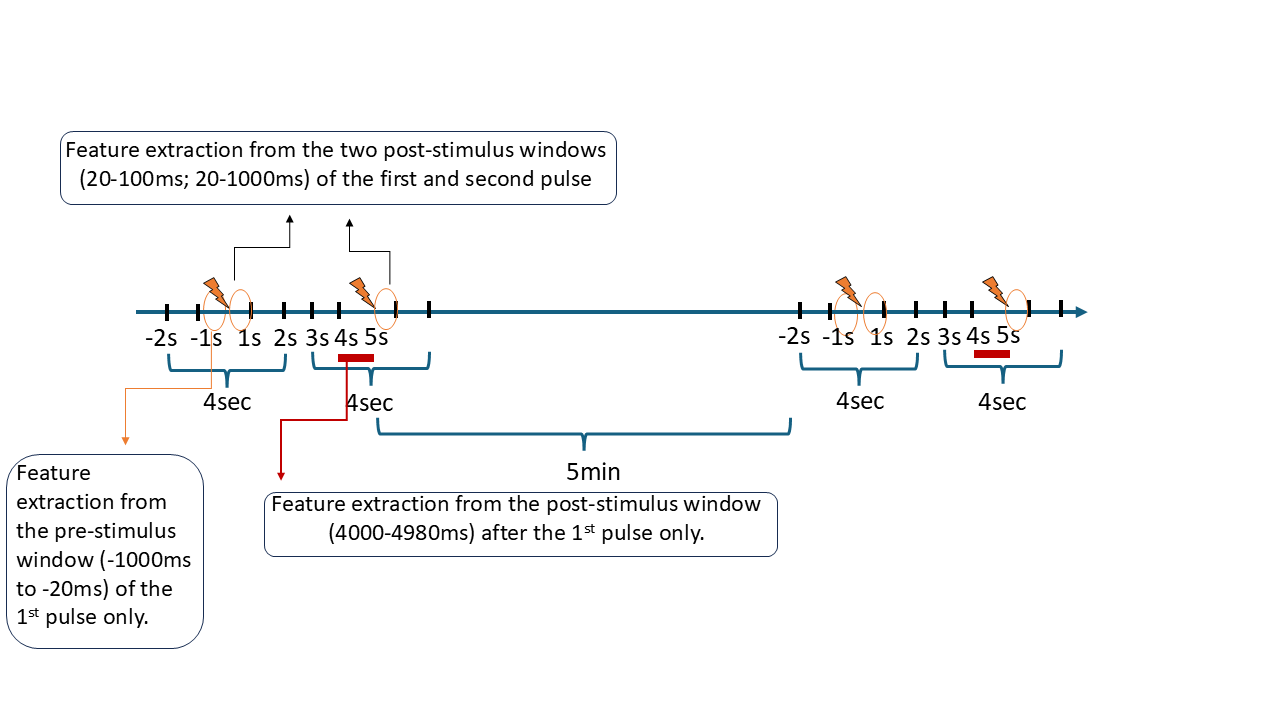


Figure S4: Schematic plot of selection of time windows relative to stimulations.


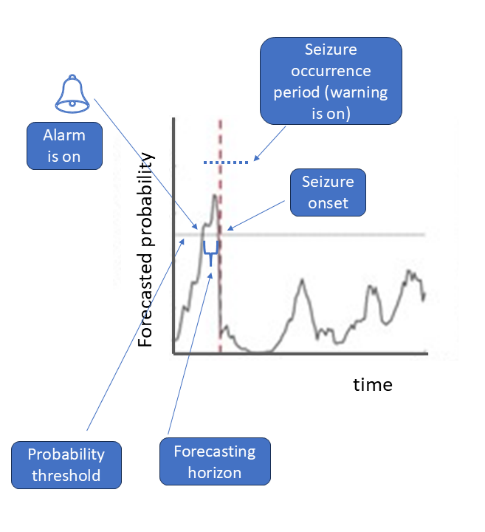


Figure S5: Illustration of the forecasting algorithm parameters in an exemplar patient.

**Supplementary Tables**

|  | KCL 1 | KCL 2 | KCL 3 | KCL 4 | KCL 5 | KCL 6 | KCL 7 | KCL 8 |
| --- | --- | --- | --- | --- | --- | --- | --- | --- |
| Sensitivity | 1 | 1 | 1 | 1 | 1 | 1 | 1 | 0 |
| IoC | 0.69 | 0.76 | 0.85 | 0.9 | 0.93 | 0.85 | 0.91 | 0 |
| Mean (IoC); standard error | 0.26; 0.024 | 0.09; 0.0059 | 0.22; 0.018 | 0.23; 0.019 | 0.29; 0.017 | 0.24; 0.037 | 0.26; 0.023 | 0; 0 |
| Tiw | 0.31 | 0.24 | 0.15 | 0.097 | 0.068 | 0.15 | 0.087 | 0 |
| Forecasting horizon (min) | 30 | 81 | 50 | 111 | 51 | 132 | 62 | N/A |
| BSS | 0.2; 0.0046 | 0.83; 0.0012 | 0.18; 0.0039 | 0.39; 0.003 | 0.35; 0.0038 | 0.12; 0.0041 | 0.35; 0.0034 | 0.23; 0.0036 |

Table S1: Forecasting values when we considered in the analysis the 4000ms to 4980ms window after the first stimulus using five signals.

|  | KCL 1 | KCL 2 | KCL 3 | KCL 4 | KCL 5 | KCL 6 | KCL 7 | KCL 8 |
| --- | --- | --- | --- | --- | --- | --- | --- | --- |
| Sensitivity | 1 | 1 | 1 | 0 | 1 | 1 | 0 | 0 |
| IoC | 0.95 | 0.77 | 0.88 | 0 | 0.84 | 0.89 | 0 | 0 |
| Mean (IoC); standard error | 0.24; 0.027 | 0.14; 0.0072 | 0.078; 0.025 | 0; 0 | 0.2; 0.012 | 0.24; 0.023 | 0; 0 | 0; 0 |
| Tiw | 0.049 | 0.23 | 0.12 | 0 | 0.16 | 0.11 | 0 | 0 |
| Forecasting horizon (min) | 36 | 97 | 50 | N/A | 104 | 105 | N/A | N/A |
| BSS | 0.22; 0.0059 | 0.74; 0.0019 | 0.19; 0.0038 | 0.14; 0.0029 | 0.71; 0.0024 | 0.43; 0.004 | 0.021; 0.0035 | 0.19; 0.003 |

Table S2: Forecasting values when we considered in the analysis the -1000ms to -20ms window before the first stimulus using five signals.

|  | KCL 1 | KCL 2 | KCL 3 | KCL 4 | KCL 5 | KCL 6 | KCL 7 | KCL 8 |
| --- | --- | --- | --- | --- | --- | --- | --- | --- |
| Sensitivity | 1 | 0 | 1 | 1 | 0 | 0 | 0 | 0 |
| IoC | 0.96 | 0 | 0.86 | 0.93 | 0 | 0 | 0 | 0 |
| Mean (IoC); standard error | 0.27; 0.024 | 0; 0 | 0.17; 0.036 | 0.22; 0.022 | 0; 0 | 0; 0 | 0; 0 | 0; 0 |
| Tiw | 0.04 | 0 | 0.14 | 0.072 | 0 | 0 | 0 | 0 |
| Forecasting horizon | 30 | N/A | 190 | 84 | N/A | N/A | N/A | N/A |
| BSS | 0.11; 0.0045 | -0.019; 0.0016 | -0.11; 0.0035 | 0.41; 0.0021 | -0.19; 0.0034 | -0.024; 0.0021 | -0.056; 0.0015 | -0.099; 0.0027 |

Table S3: Forecasting values when we considered in the analysis the 4000ms to 4980ms window after the first stimulus using one signal.

|  | KCL 1 | KCL 2 | KCL 3 | KCL 4 | KCL 5 | KCL 6 | KCL 7 | KCL 8 |
| --- | --- | --- | --- | --- | --- | --- | --- | --- |
| Sensitivity | 1 | 1 | 1 | 0 | 0 | 0 | 0 | 0 |
| IoC | 0.98 | 0.82 | 0.95 | 0 | 0 | 0 | 0 | 0 |
| Mean (IoC); standard error | 0.26; 0.034 | 0.14; 0.0079 | 0.23; 0.023 | 0; 0 | 0; 0 | 0; 0 | 0; 0 | 0; 0 |
| Tiw | 0.02 | 0.18 | 0.049 | 0 | 0 | 0 | 0 | 0 |
| Forecasting horizon (min) | 19 | 27 | 72 | N/A | N/A | N/A | N/A | N/A |
| BSS | 0.0027; 0.0055 | 0.88; 0.0009 | 0.72; 0.0012 | -0.16; 0.0069 | -0.17; 0.0032 | -0.032; 0.0027 | 0.11; 0.0025 | -0.034; 0.0028 |

Table S4: Forecasting values when we considered in the analysis the 4000ms to 4980ms window after the first stimulus using all signals.

|  | KCL 1 | KCL 2 | KCL 3 | KCL 4 | KCL 5 | KCL 6 | KCL 7 | KCL 8 |
| --- | --- | --- | --- | --- | --- | --- | --- | --- |
| Sensitivity | 0 | 0 | 1 | 0 | 0 | 0 | 0 | 0 |
| IoC | 0 | 0 | 0.85 | 0 | 0 | 0 | 0 | 0 |
| Mean (IoC); standard error | 0; 0 | 0; 0 | 0.21; 0.028 | 0 | 0 | 0 | 0 | 0 |
| Tiw | 0 | 0 | 0.15 | 0 | 0 | 0 | 0 | 0 |
| Forecasting horizon | N/A | N/A | 212 | N/A | N/A | N/A | N/A | N/A |
| BSS | -0.02; 0.0015 | -0.032; 0.002 | -0.26; 0.0052 | -0.12; 0.0024 | -0.15; 0.0032 | -0.057; 0.0022 | -0.015; 0.0023 | -0.11; 0.0038 |

Table S5: Forecasting values when we considered in the analysis the -1000ms to -20ms window before the first stimulus using one signal.

|  | KCL 1 | KCL 2 | KCL 3 | KCL 4 | KCL 5 | KCL 6 | KCL 7 | KCL 8 |
| --- | --- | --- | --- | --- | --- | --- | --- | --- |
| Sensitivity | 1 | 1 | 1 | 0 | 0 | 0 | 0 | 0 |
| IoC | 0.99 | 0.82 | 0.95 | 0 | 0 | 0 | 0 | 0 |
| Mean (IoC); standard error | 0.16; 0.034 | 0.14; 0.0095 | 0.24; 0.03 | 0; 0 | 0; 0 | 0; 0 | 0; 0 | 0; 0 |
| Tiw | 0.0098 | 0.18 | 0.048 | 0 | 0 | 0 | 0 | 0 |
| Forecasting horizon (min) | 14 | 27 | 72 | N/A | N/A | N/A | N/A | N/A |
| BSS | 0.028; 0.0043 | 0.85; 0.0009 | 0.65; 0.0017 | 0.061; 0.0035 | -0.14; 0.0024 | -0.13; 0.004 | 0.02; 0.0021 | -0.14; 0.0034 |

Table S6: Forecasting values when we considered in the analysis the -1000ms to -20ms window before the first stimulus using all signals.
